# Comparative Study of Nutritional Composition, Physiological Indicators, and Genetic Diversity in *Litopenaeus vannamei* from Different Aquaculture Populations

**DOI:** 10.3390/biology13090722

**Published:** 2024-09-14

**Authors:** Yundong Li, Siyao Cao, Shigui Jiang, Jianhua Huang, Qibin Yang, Song Jiang, Lishi Yang, Falin Zhou

**Affiliations:** 1 South China Sea Fisheries Research Institute, Chinese Academy of Fishery Sciences, Key Laboratory of South China Sea Fishery Resources Exploitation and Utilization, Ministry of Agriculture and Rural Affairs, Guangzhou 510300, China; liyd2019@163.com (Y.L.); cao13793933909@163.com (S.C.); jiangsg@21cn.com (S.J.); huangjianhua@scsfri.ac.cn (J.H.); yangqibin1208@163.com (Q.Y.); tojiangsong@163.com (S.J.); yangls2016@163.com (L.Y.); 2 College of Fisheries and Life Sciences, Dalian Ocean University, Dalian 116023, China; 3 Key Laboratory of Efficient Utilization and Processing of Marine Fishery Resources of Hainan Province, Sanya Tropical Fisheries Research Institute, Sanya 572018, China; 4 Shenzhen Base of South China Sea Fisheries Research Institute, Chinese Academy of Fishery Sciences, Shenzhen 518108, China

**Keywords:** *Litopenaeus vannamei*, germplasm resources, population, genetic diversity

## Abstract

**Simple Summary:**

This study investigates the nutritional quality and genetic diversity of Pacific white shrimp (*Litopenaeus vannamei*) from three different aquaculture farms in Guangdong Province, China. We assessed the shrimp’s muscle tissue for key nutrients like protein, fat, and amino acids, alongside a detailed genetic analysis. Our findings reveal that the nutritional content is consistent across all three populations, indicating the reliable protein quality and essential nutrient levels critical for shrimp health and growth. However, significant differences in genetic diversity were observed, with one population exhibiting reduced genetic diversity and signs of inbreeding. These results underscore the necessity of implementing effective breeding strategies to enhance genetic diversity and ensure the sustainable development of *L. vannamei* in aquaculture. This study provides essential insights for optimizing breeding practices to maintain the long-term health and productivity of this vital species in the aquaculture industry.

**Abstract:**

This study aimed to evaluate the quality and genetic diversity of farmed *Litopenaeus vannamei* across three distinct populations from Maoming City (MM), Zhanjiang City (ZJ), and Yangjiang City (YJ) in Guangdong Province. Muscle tissues from *L. vannamei* were analyzed for phenotypic traits, conventional nutrients, amino acids, and fatty acids, while genetic diversity was assessed using whole genome sequencing techniques. The analysis revealed that the crude protein content in shrimp across the three populations ranged from 20.87 to 21.95 g/100 g, crude fat content ranged from 0.90 to 1.50 g/100 g, essential amino acid content ranged from 5.55 to 5.86 g/100 g, total amino acid content ranged from 14.73 to 15.27 g/100 g, total fatty acid content ranged from 682.73 to 793.97 mg/100 g, total antioxidant capacity (T-AOC) ranged from 2.68 to 2.72 μmol/g, superoxide dismutase (SOD) activity ranged from 1021.97 to 1057.21 U/g, and catalase (CAT) activity ranged from 78.65 to 81.33 μmoL/min. No significant differences were observed in ash and crude fat levels among conventional nutrients, nor in the biochemical indexes T-AOC, CAT, and SOD. Genetic analysis showed that the single nucleotide polymorphism density (SNP/Kb) ranged from 15.323 to 17.461, nucleotide diversity (π) ranged from 2.98 × 10^−5^ to 15.84 × 10^−5^, polymorphism information content (PIC) ranged from 0.300 to 0.317, heterozygosity (Ho) ranged from 0.033 to 0.048, and inbreeding coefficients (FIS) ranged from 0.834 to 0.887. The genetic differentiation index (FST) values among the three populations ranged from 0.056 to 0.106. This study provides an evaluation of the germplasm resources and genetic diversity of farmed *L. vannamei*, offering insights for the efficient management and sustainable utilization of this species’ germplasm resources.

## 1. Introduction

*Litopenaeus vannamei*, classified within the phylum Arthropoda, subphylum Crustacea, class Malacostraca, order Decapoda, and family Penaeidae, is a species of considerable global importance in aquaculture [1]. Known as the Pacific white shrimp, it is highly valued for its rapid growth and broad environmental adaptability, making it a cornerstone of the aquaculture industry [2]. Although *L. vannamei* is noted for its growth rate and environmental adaptability, it does not possess uniform resistance to all diseases. In practice, this species remains vulnerable to various diseases, which can significantly impact its growth and production efficiency [3]. However, the domestic aquaculture environment has deteriorated, leading to frequent disease outbreaks [4]. Concurrently, the expansion of aquaculture scale and the indiscriminate selection of shrimp broodstock—often directly from ponds—have resulted in reduced genetic diversity and inbreeding depression [5]. These practices have decreased production efficiency, severely impacting the conservation and utilization of superior genetic resources and overall production development [6]. Therefore, while breeding strategies aim to improve production efficiency, they also raise concerns about diminishing genetic diversity within shrimp populations.

Genetic diversity is fundamental for maintaining species adaptability and long-term survival and is a critical criterion for evaluating natural biological resources. Low genetic diversity can result in inbreeding depression, characterized by slow growth, reduced immunity, and increased susceptibility to diseases [7]. In the context of commercial *L. vannamei* farming, excessive reliance on a limited number of genotypes can reduce the genetic diversity of the entire population, thereby increasing vulnerability to environmental changes and disease outbreaks. Ensuring the sustainable development of *L. vannamei* aquaculture requires balancing breeding practices with the conservation of genetic diversity to maintain robust and adaptable shrimp populations [8].

This study aims to compare the phenotypic differences, nutritional composition, physiological–biochemical indicators, and genetic diversity among three different populations of *L. vannamei* using whole-genome sequencing technology. By examining the muscle nutritional components and physiological–biochemical indexes, we aim to understand how genetic diversity influences their growth and health. This comprehensive analysis seeks to elucidate the genetic, nutritional, and physiological differences among these populations, providing scientific evidence for future breeding programs to ensure the sustainable development of the *L. vannamei* aquaculture industry. Furthermore, the findings offer new perspectives and methods to optimize farming practices, thereby enhancing the efficiency of *L. vannamei* cultivation. This research also serves as a reference for other aquaculture species’ genetic diversity conservation and breeding strategies.

## 2. Materials and Methods

### 2.1. Test Materials

**Figure 1:**
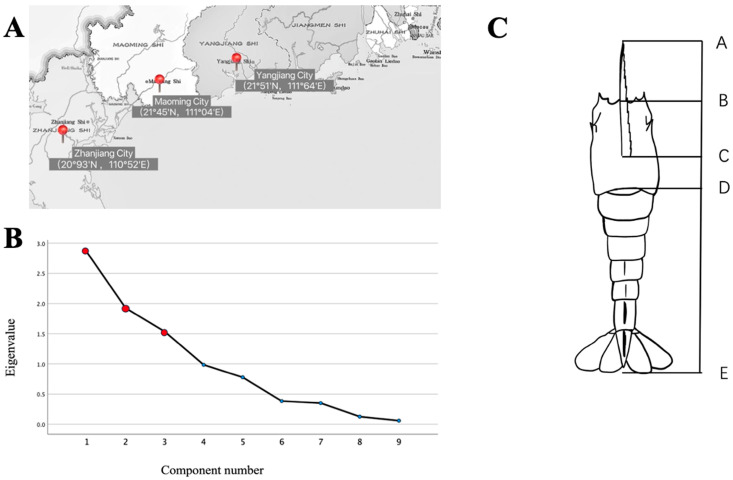
Illustration of the study area and data analysis. (**A**) Geographic location of the three sampling sites for *Litopenaeus vannamei*. (**B**) Principal component analysis of eight characteristics of three populations of *Litopenaeus vannamei* in a gravel map (red dots represent three principal components with characteristic values greater than 1). (**C**) Schematic diagram of the measurement of some morphological characteristics of *Litopenaeus vannamei*. AC: frontal angle length (FAL); BD: cephalothorax length (CL); BE: body length (BL); AE: total length (TL).

In September 2022, a total of three geographically distinct populations of cultured *L. vannamei* were sampled from representative aquaculture farms in Southeast China. Specifically, 30 samples were collected from each of the following locations: Maoming City (LvA), Zhanjiang City (LvB), and Yangjiang City (LvC). For simplicity, these populations are identified by letter codes corresponding to their collection sites. The sampling locations and their corresponding codes are illustrated in Figure 1A. Detailed data regarding the cultured *L. vannamei* samples from each site are provided in Table 1.

The three farms employ distinct aquaculture practices tailored to their specific environments. The Maoming farm (LvA) utilizes open-air earthen ponds, drawing seawater from the nearby Beibu Gulf. This farm focuses on maintaining moderate stocking densities from 30–35 initially to 10–15 shrimp per square meter during the growth phase, and provides high-protein (35–40%) feed with 5–8% fat and 15–18% energy content, sourced mainly from fish meal, soybean meal, and lecithin to support optimal growth conditions. In contrast, the Zhanjiang farm (LvB) operates a semi-closed recirculating system with concrete ponds, maintaining lower stocking densities ranging from 25–30 initially to 8–12 shrimp per square meter. It uses an automated feeding system with imported feed consisting of 40% protein, 8–10% fat, and 18–20% energy to ensure consistent shrimp quality. The Yangjiang farm (LvC) employs modern plastic-lined ponds, utilizing seawater treated through natural sedimentation. This farm emphasizes controlled stocking densities from 20–25 initially to 5–10 shrimp per square meter and uses nutrient-enriched feed with 40% protein, 10–12% fat, and 20–22% energy, including high proportions of fish meal, soybean meal, lecithin, and probiotics to promote uniform growth. All farms implement stringent parent shrimp management practices, sourcing shrimp from diverse genetic pools to avoid inbreeding, with comprehensive monitoring and documentation to ensure that genetic diversity is maintained.

### 2.2. Measurement Methods

The shrimp were weighed accurately using an electronic balance (accuracy of 0.01 g). Eighty-two phenotypic traits of shrimp were measured accurately using vernier calipers (accuracy of 0.01 mm) (Figure 1B). These traits included total length (TL), body length (BL), cephalothorax length (CL), frontal angle length (FAL), number of frontal teeth (NFT), length of the second leg (LSL), and the distance between the bases of the fifth step foot (Step 5 Interbasal distance, SID). Measurements such as the first touch (FT) and the second whip (SW) were also recorded. The surface moisture of the samples was absorbed by gauze before weighing [9]. The measurement of phenotypic traits in *L. vannamei* was conducted to assess physical and morphological differences among the three populations. These traits, including total length and body length, serve as indicators of growth performance and health. By analyzing these characteristics, we aim to link phenotypic variation with genetic diversity and environmental factors, providing insights into the factors that influence shrimp quality and development in aquaculture.

### 2.3. Biochemical Composition Analysis

The abdominal muscles of the shrimp were taken to determine the moisture, crude ash, crude protein, crude fat, and total sugar contents in the muscles. Moisture was determined by the electrothermal constant temperature drying method (GB5009.3-2016) [10]; ash was determined by the Mafu furnace ashing method (GB/T6438-92) [11]; crude protein was determined by the Kjeldahl nitrogen determination method (GB/T6432-94) [12]. Crude fat was determined by the acid hydrolysis test method (GB5009.6-2016) [13]. Total sugar was determined by ultraviolet–visible spectrophotometry (GB/T9695.31-2008) [14]. Apart from the moisture content, the specific determination steps of the remaining indicators followed the AOAC guidelines (1984).

### 2.4. Nutritional Quality Assessment

Seventeen common amino acids were determined by the hydrochloric acid hydrolysis method (GB/T5009.124-2016) [15]. The Hitachi LA8080 amino acid autoanalyzer(Hitachi High-Tech Corporation, Tokyo, Japan) determined the amino acid content, and tryptophan was not determined because it was des I'm sure to keep ittroyed in the process of hydrolysis. The fatty acid profile was determined by gas chromatography (GB/T5009.168-2016) [16].

### 2.5. Antioxidant Enzyme Activity Assays

The abdominal muscles of the shrimp were used to determine the activities of superoxide dismutase (SOD), catalase (CAT), and total antioxidant capacity.

Superoxide dismutase (SOD) activity is defined as the amount of enzyme per milliliter of reaction solution corresponding to 50% inhibition of superoxide dismutase as one unit of enzyme activity (U/mg). This indicator was determined by referring to the instruction manual of the WST-8 method activity assay kit(JianCheng, Nanjing, China) [17]. The activity of the WST-8 method was determined according to the instructions for the WST-8 Activity Kit.

Catalase (CAT) activity was defined as one unit of enzyme activity (U/mg) per milliliter of tissue protein breaking down 1 μmol of H_2_O_2_ per minute.

Total antioxidant capacity (T-AOC) is defined as the total antioxidant level consisting of various antioxidant substances, antioxidant enzymes, etc. This index was determined by referring to the instructions of the total antioxidant capacity (T-AOC) assay kit using the ABTS method.

### 2.6. Genetic Diversity

Genomic DNA was extracted from the tail muscle of *L. vannamei* using the phenol-chloroform method. DNA integrity was assessed via 1% agarose gel electrophoresis, while concentration and purity were measured using UV spectrophotometry(Shimadzu Corporation, Kyoto, Japan), ensuring that DNA concentration exceeded 12.5 ng/µL. For sequencing library preparation, the DNA was fragmented into smaller pieces using sonication. Libraries were then constructed according to the Illumina TruSeq protocol, which involved end-repair to blunt the DNA ends, A-tailing to add an adenine overhang, adapter ligation to attach sequencing adapters, and PCR amplification to enrich the library. The quality and quantity of the constructed libraries were evaluated using a bioanalyzer and quantitative PCR (qPCR) to ensure suitability for sequencing.

High-throughput sequencing was performed on the Illumina platform(Illumina, Inc., San Diego, CA, USA), generating raw reads. Initial quality control of these reads was conducted using FastQC to identify any sequencing biases or errors. The reads were aligned to the reference genome using the Burrows–Wheeler Aligner (BWA)(Wellcome Trust Sanger Institute, Cambridge, UK), an efficient and accurate alignment tool [18]. Variant calling was performed using the Genome Analysis Toolkit (GATK)(Broad Institute, Cambridge, MA, USA), which identified single nucleotide polymorphisms (SNPs) and insertions/deletions (indels), producing an initial Variant Call Format (VCF) file [19]. To ensure the accuracy of the variant data, the VCF file was filtered using VCF tools, applying stringent criteria for variant quality and coverage depth to exclude low-confidence variants. The remaining high-confidence variants were functionally annotated using ANNOVAR(University of Southern California, Los Angeles, CA, USA), providing insights into the potential effects of these variants on gene function [20]. For population genetic analysis, genotype data underwent further quality control using PLINK(Broad Institute, Cambridge, MA, USA), with checks for missing data, minor allele frequency thresholds, and Hardy–Weinberg equilibrium [21]. Population structure was analyzed using ADMIXTURE(Broad Institute, Cambridge, MA, USA) and Principal Component Analysis (PCA) to identify genetic clusters and assess potential population stratification. Linkage disequilibrium (LD) was calculated using Haploview, offering insights into the genetic linkage between markers [22]. Selection signals were detected using iHS (Integrated Haplotype Score)( Pritchard Lab, University of Chicago, Chicago, IL, USA) and XP-EHH (Cross-population Extended Haplotype Homozygosity) (Sabeti Lab, Harvard University, Cambridge, MA, USA) methods [23].

### 2.7. Data Analysis

The experimental data were expressed as mean ± standard deviation, and one-way ANOVA (one-way ANOVA) was performed with SPSS 27.0 statistical software, and Duncan’s multiple comparisons analysis was performed if there were significant differences (*p* < 0.05) [24]. The proportional parameters were input into SPSS 27.0 statistical software to obtain the contribution rate and cumulative contribution rate of each principal component through the factor analysis of SPSS, and then to obtain which of the nine proportional parameters played a decisive role [25]; using the DNASP5.0 software, the analysis of nucleotide diversity (π) was computed [26]; the PopGene32 software was used to calculate the completion of the observed heterozygosity (Ho) [27]; and the polymorphic information content (PIC) of each locus in each population was calculated using PIC_CALC0.6 software [28]. The inbreeding coefficient (FIS) and genetic differentiation index (FST) were carried out using Arlequin3.5 [29]; gene flow (Nm) was calculated according to the formula: Nm = 0.25 (1 − FST)/FST [30], and the genetic distance between populations (DR) was calculated according to the formula: −In (1 − FST) [31].

## 3. Results

### 3.1. Analysis of Phenotypic Traits

A one-way ANOVA was conducted to analyze eight morphometric traits across three groups of *L. vannamei* (Table 2). The results showed variations in these traits among the populations, but none of the differences were statistically significant (*p* > 0.05). Notably, the second whip (SW) length and cephalothorax length (CL) of the LvB population were markedly larger than those of the other populations, with the LvA population exhibiting the smallest values. In the Principal Component Analysis (Table 3), it was noted that the initial principal component accounted for 31.756% of the variance, predominantly representing metrics such as body length (BL) about the length of the second leg (LSL), body length (BL) compared to the distance between the bases of the fifth step foot (SID), body length (BL) juxtaposed with cephalothorax length (CL), and body length (BL) relative to frontal angle length (FAL). This refers to principal component 1 primarily reflecting the morphological aspects associated with body length (BL), suggesting considerable morphological distinctions across the three *L. vannamei* populations. The second principal component contributed 21.383% to the variation, signifying a focus on metrics like the first touch (FT) length per cephalothorax length (CL), the first touch (FT) length in relation to body length (BL), and other relevant parameters. Thus, principal component 2 predominantly highlights the morphological characteristics of the cephalothorax length (CL), suggesting that differences in this area significantly contribute to the overall morphological variations observed among the three populations. The third principal component, responsible for 17.065% of the variation, predominantly emphasizes metrics such as the second whip (SW) length per cephalothorax length (CL) and the second whip (SW) length relative to body length (BL). Principal component 3 primarily mirrors the morphological attributes associated with the second whip (SW) length. The cumulative contribution of the three principal components amounts to 70.204%. Notably, the three principal components with eigenvalues surpassing 1 were derived from the nine indicators in Figure 1C. These findings highlight that body length (BL), carapace length (CL), and second whip (SW) length vary significantly among *L. vannamei* populations. These morphological features are potentially important in distinguishing populations, suggesting that they may be important clues for the further investigation of genetic and ecological differences among populations.

### 3.2. Conventional Nutrients

**Figure 2:**
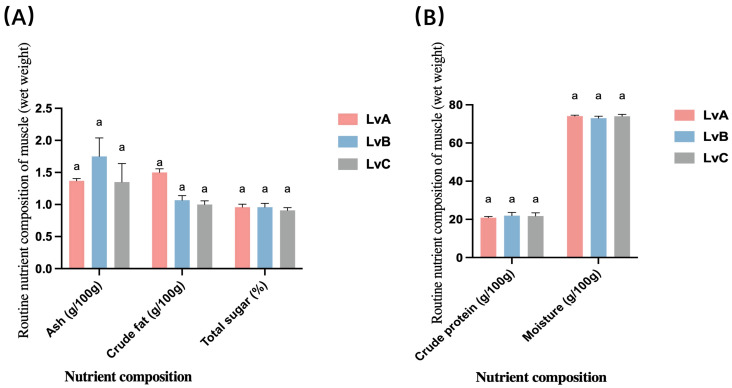
Routine nutrient composition of muscle (wet weight) of three groups of *Litopenaeus vannamei*. (**A**) The contents of ash, crude fat, and total sugar in three populations. (**B**) The water content and crude protein content of the three populations. Superscript “a” indicates no statistically significant difference (*p* > 0.05) between the groups.

Fresh muscle samples´ protein and fat contents from *L. vannamei* stocks LvA, LvB, and LvC were analyzed (Figure 2). LvA exhibited a crude protein content of 20.87 ± 0.64 g/100 g and a crude fat content of 1.50 ± 0.10 g/100 g. LvB showed a slightly higher protein content at 21.95 ± 1.65 g/100 g and a lower fat content of 1.05 ± 0.15 g/100 g. LvC had a protein content of 21.75 ± 1.70 g/100 g and a fat content of 0.90 ± 0.10 g/100 g. The conventional nutrient contents, including crude ash and fat, were consistent among the three stocks, with no significant differences observed (*p* > 0.05). This suggests that, despite minor variations in protein and fat content, the overall nutritional composition across the three populations remains comparable, with no statistically significant differences observed.

### 3.3. Amino Acid Composition and Content

Our comprehensive analysis detected a total of 17 amino acids in the three populations of *L*. *vannamei*, reflecting a thorough assessment of the species’ amino acid profile. This detailed analysis included seven essential amino acids (EAA: Thr, Val, Met, Phe, Ile, Leu, Lys), although tryptophan was not detected due to disruption during acid hydrolysis. Additionally, two semi-essential amino acids (SEAA: His, Arg) and eight non-essential amino acids (NEAA: Asp, Ser, Glu, Gly, Ala, Cys, Pro, Tyr) were identified (Table 4). This comprehensive dataset provides a wealth of information on the amino acid composition of *L. vannamei*, offering valuable insights for understanding its nutritional quality and supporting further research on its aquaculture management and breeding.

### 3.4. Fatty Acid Composition and Content

A total of 16 fatty acids were detected in the three populations of *L. vannamei*, including six saturated fatty acids (SFA), two monounsaturated fatty acids (MUFA), and five polyunsaturated fatty acids (PUFA) (Table 5). The total content of saturated fatty acids ranged from 267.17 to 317.83 mg/100 g; the total content of monounsaturated fatty acids ranged from 10.43 to 12.53 mg/100 g; and the total content of polyunsaturated fatty acids ranged from 242.42 to 277.10 mg/100 g.

### 3.5. Analysis of Physiological Indicators

**Figure 3:**
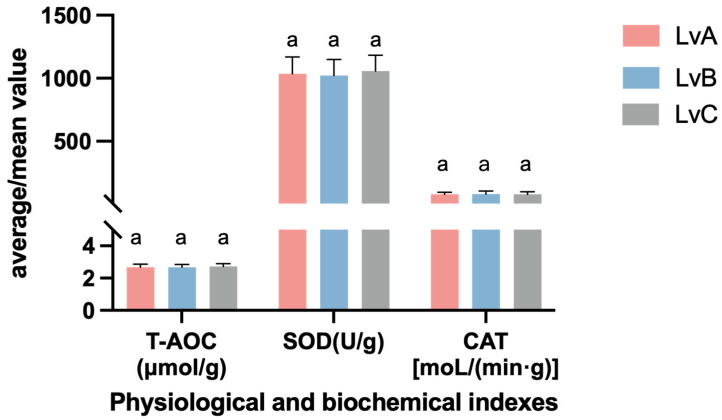
Physiological and biochemical indexes of the three populations of *Litopenaeus vannamei.* Superscript “a” indicates no statistically significant difference (*p* > 0.05) between the groups.

The total antioxidant capacity (T-AOC) of *L. vannamei* LvA was 2.68 ± 0.19 μMol/g; the activity range of superoxide dismutase (SOD) was 1035.72 ± 132.34 U/g; the activity range of catalase (CAT) was 78.65 ± 17.49 μMol/L/(ming). The total antioxidant capacity (T-AOC) of LvB was 2.68 ± 0.17 μMol/g; the activity range of superoxide dismutase (SOD) was 1021.97 ± 127.25 U/g; the activity range of catalase (CAT) was 81.33 ± 23.52 μMol/L/(ming). The total antioxidant capacity (T-AOC) of LvC was 2.72 ± 0.18 μMol/g; the activity range of superoxide dismutase (SOD) was 1057.21 ± 126.62 U/g; the activity range of catalase (CAT) was 80.14 ± 19.55 μMol/L/(ming). There were no significant differences in physiological and biochemical indicators among the groups (Figure 3).

### 3.6. Genetic Diversity and Population Structure Analysis

#### Statistical Analysis of Genetic Diversity Parameters

In an in-depth assessment of the genetic diversity of the three populations of *L. vannamei* (LvA, LvB, and LvC) (Table 6), we found significant differences in SNP density and nucleotide diversity among the three populations. Although the observed heterozygosity was relatively consistent among the three, the nucleotide diversity of LvC (15.84 × 10^−5^) was significantly higher than that of LvA (3.11 × 10^−5^) and LvB (2.98 × 10^−5^), suggesting that the LvC population may have richer genetic diversity.

In the analysis of genetic differentiation among populations (Table 7), the genetic differentiation index between LvB and LvC (FST = 0.106) was the highest among the three, indicating that there was a large genetic isolation between the two populations. In contrast, the genetic connection between LvA and LvB was relatively closer (FST = 0.056). In addition, gene flow analysis further revealed genetic isolation between populations, especially the lowest gene flow observed between LvB and LvC (Nm = 2.108). These findings highlight the need to strengthen genetic monitoring of *L. vannamei* populations to ensure the diversity of genetic resources and the maintenance of population health, especially in the face of dual environmental and economic pressures. Through the effective management and use of these genetic data, we can lay a solid foundation for future breeding activities.

## 4. Discussion

### 4.1. Analysis of Differences in Phenotypic Traits of Different Populations of Litopenaeus vannamei

The results of the morphometric analysis of *L. vannamei* suggest a high degree of uniformity in the measured proportions among the different groups. Significant morphological differences may not be present due to the groups being cultivated under similar aquaculture conditions. Additionally, selective breeding strategies might have been consistent, focusing on common important traits such as growth, which could reduce morphological differences among the groups.

Although of morphometric proportions did not reveal significant differences, principal component analysis (PCA) was applied to the three groups of *L. vannamei* and successfully identified three main components. These components predominantly reflect the shrimp’s total and second antennal lengths. These findings indicate that these traits play a significant role in distinguishing between shrimp groups and can be considered important discriminators. Total length, as a fundamental indicator of growth and development, directly reflects the health and growth rate of an individual. Carapace length is not only crucial for morphological assessment but also relates to predation behavior and immune function. The length of the second antenna plays a key role in the sensory and locomotive abilities of shrimp, potentially affecting their adaptability and behavioral performance. The cumulative contribution of these three principal components was 70.20%, similar to the results of Li Xiaoshuang et al. [32], who reported a cumulative contribution rate of 72.58% from four principal components in a morphological variation analysis of six groups of *L. vannamei.* This similarity suggests that these components can explain a significant portion of the morphological variation and are highly representative. These traits can be utilized not only to distinguish between these three groups but also in identifying other populations. Therefore, future breeding and management efforts should integrate genetic diversity with key morphological traits. By ensuring breeding objectives are met while enhancing genetic diversity protection, sustainable development and the health of *L. vannamei* populations can be ensured.

### 4.2. Analysis of Differences in the Routine Nutrient Composition of the Muscles of L. vannamei from Different Stocks

The evaluation of the nutritional value of aquatic products often relies on assessing the protein and fat content present in the muscle tissue [17]. In this study, the research findings indicate that the average crude protein content in the muscle tissue of *L. vannamei* across three populations exceeds 21.52%, with no significant differences observed between them. Comparatively, this protein content surpasses that of *Mozambican Penaeus Monodon* (18.67%), *Macrobrachium rosenbergii* (18.27%) [33], *Marsupenaeus japonics* (17.74%) [34], *M. nipponenses* (17.71%), and *Exopalaemon modestus* (17.60%) [35]. These results align with the research conducted by Chen Xiaohan et al. [36] in 2001, showcasing *L. vannamei*’s protein content of 21.57%. Furthermore, the fat content in the three populations of *L. vannamei* falls within the range of 0.90% to 1.50%, consistent with *Exopalaemon modestus* (1.43%) [35] and *Macrobrachium nipponense* (1.19%) [34]. However, it is lower than *Marsupenaeus japonics* (2.08%) [34], *Macrobrachium rosenbergii* (1.97%) [33], and *Cambarus clarkii* (1.70%) [37]. The lower fat content of *L. vannamei* positively influences its acceptance among consumers, as low-fat content is generally perceived as healthier. This finding highlights the unique nutritional profile of *L. vannamei*, further emphasizing its value in commercial aquaculture. The study demonstrates that *L. vannamei* has significant advantages in terms of muscle crude protein and fat contents, particularly its superior protein content, enhancing its market competitiveness. These findings not only help elevate the status of *L. vannamei* in the aquaculture industry but also provide essential scientific evidence for future selective breeding and nutritional improvement. This study showed no statistically significant differences in the conventional nutritional components across the three muscle groups (*p* > 0.05). This may be related to the uniformity of feed provided during the cultivation process [38]. Future research should continue to focus on the variations in the nutritional composition of *L. vannamei* under different environmental and feeding conditions to optimize farming strategies and maximize its nutritional and economic value.

### 4.3. Analysis of Differences in Muscle Amino Acid Composition of Different Stocks of Litopenaeus vannamei

Most aquatic animals contain high-quality animal proteins [39]. Protein quality mainly depends on the protein content, essential amino acid content, and the proportion and digestibility and the type and content of amino acids play an important role in the growth and development of shrimp as well as in the evaluation of their quality, which is the main factor determining the nutritional value [40,41]. This study analyzes the amino acid content of three populations of *L. vannamei*, revealing no significant differences among the populations. This indicates a high degree of consistency in amino acid composition across different populations, despite certain genetic variations and environmental differences. Such consistency may stem from a similar genetic basis among the populations, particularly concerning genes related to amino acid metabolism, and from cultivation under similar conditions and feed formulations. Consequently, the environmental impact on amino acid composition is minimal [42]. This stability is crucial for ensuring that *L. vannamei* consistently provides high-quality protein across different aquaculture environments.

According to the standards for high-quality food protein set by the Food and Agriculture Organization of the United Nations (FAO) and the World Health Organization (WHO) [43], good-quality proteins should not only contain a complete range of essential amino acids but also have the right or correct proportion of essential amino acids to each other [44,45]. The content and proportion of essential amino acids are used as indicators. The nutritional value of shrimp protein was evaluated using the content and ratio of essential amino acids as indicators. According to the essential amino acid model recommended by FAO/WHO, the ratio of EAAs/TAAs should be greater than or equal to 0.40, and the EAAs/NEAAs ratio should be greater than or equal to 0.06 [46]. It can be determined from Table 5 that the ratio of EAAs/TAAs in shrimp from *L. vannamei* was 0.37–0.38, which was very close to 0.40, and the ratio of EAAs/NEAAs in shrimp was 0.70–0.72, which was relatively close to 0.60. These results suggest that the high-quality protein in shrimp meat or by-products is associated with the ratios of essential amino acids to total amino acids (EAAs/TAAs) and essential amino acids to non-essential amino acids (EAAs/NEAAs). *L. vannamei* exhibits the advantage of high-quality protein and a balanced amino acid composition, facilitating efficient protein synthesis and growth. This amino acid balance is crucial for enhancing shrimp growth performance and health, reinforcing its status as a premium aquaculture product [47].

### 4.4. Analysis of Differences in Muscle Fatty Acid Composition of Different Populations of Litopenaeus vannamei

The composition and content of fatty acids in food is an essential indicator for evaluating the nutritional value of lipids [46]. Saturated fatty acids play an important role in the energy metabolism of shrimp. However, humans’ excessive intake of saturated fatty acids is detrimental to human health and may increase the risk of cardiovascular disease. Oleic acid in monounsaturated fatty acids is also a major source of energy in the growth and metabolism of shrimp [46]. Polyunsaturated fatty acids (PUFAs) are bioactive substances with unique physiological functions, and their nutritional value is positively correlated with the content of unsaturated fatty acids [46]. The nutritional value of PUFAs is positively correlated with their content [48].

This study conducted a comparative analysis of the fatty acid composition in three populations of *L. vannamei* (LvA, LvB, and LvC). Significant differences were observed among the populations regarding total fatty acid content and specific fatty acid types. The LvA population exhibited the highest total fatty acid content, particularly in polyunsaturated fatty acids (PUFAs) and essential omega-3 fatty acids, such as eicosapentaenoic acid (EPA) and docosahexaenoic acid (DHA). These fatty acids are crucial for the growth and health of *L. vannamei* and significantly enhance their nutritional value. The content of n-3 polyunsaturated fatty acids (PUFA) ranged from 109.87 to 124.43 mg/100 g, and the content of n-6 polyunsaturated fatty acid (PUFA) ranged from 165.93 to 192.13 mg/100 g. Significant differences (*p* < 0.05) were observed in C16:0, C18:1n9c, C22:1n9, and C20:5n3 among the different groups. These findings illustrate the diversity in fatty acid profiles among the studied populations, potentially reflecting variations in their diet, genetic makeup, and environmental conditions. In particular, the LvA population had the highest total content of polyunsaturated fatty acids (PUFAs) and essential omega-3 fatty acids such as EPA and DHA, which are essential for shrimp growth and health, significantly increasing their nutritional value. A possible explanation is that LvA populations live in lagoons with natural substrates and have access to a more diverse range of natural food resources. Combined with the higher proportions of fishmeal, soybean meal, and phospholipids in their feed, these rich nutrient sources promote the synthesis of key healthy fatty acids such as EPA and DHA. This comprehensive feed and natural food supply not only provides balanced nutrition, but also contributes to the diversity and enrichment of fatty acids.

In contrast, the LvB and LvC populations were in an environment with artificial concrete and canvas substrates. Although the crude protein and energy content of the feed was higher, the limitations of natural food may have affected the synthesis of specific fatty acids, especially polyunsaturated fatty acid content. This difference in environment and feed may result in these populations being lower in key fatty acids than LvA. Although high fat and energy in the diet is beneficial for the rapid growth of shrimp, this is not necessarily effective in promoting the production of essential fatty acids, indicating that optimizing the quality and quantity of fatty acids is critical. The contents of EPA and DHA in the muscle of the *Marsupenaeus japonicas* accounted for 1.70% and 10.92% of the total fatty acid content, respectively. In the present study, the muscle fatty acid contents of EPA, DHA, and EPA + DHA were higher in *L. vannamei*, ranging from 6.9 to 7.1%, 7.6 to 8.4%, and 14.49 to 15.56%, respectively, which were significantly higher than those of shrimp species such as *M. nipponenses* (EPA0.05%), *Exopalaemon modestus* (EPA0.04%), and *Monopterus albus* (EPA 0.74%, DHA 1.33%) and *snakehead* (DHA 1.4%) [49]. As a result, *L. vannamei* is a high-quality shrimp with a perfect fatty acid profile. Although the amino acid composition of the three populations of *L. vannamei* is consistent, the difference in fatty acid composition provides a direction for optimizing aquaculture and nutrition management strategies. Breeding environment and feed formula play a key role in optimizing the fatty acid composition of shrimp. The future aquaculture management and nutrition strategy should focus on improving the nutritional value and market competitiveness of shrimp by adjusting the key ingredients in the feed and optimizing environmental conditions. The scientific design of feed nutrition and comprehensive consideration of natural and artificial food sources will help to improve the health level and nutritional quality of shrimp products. In addition, by increasing the contents of polyunsaturated fatty acids (PUFAs) and omega-3 fatty acids, the nutritional status of vulnerable groups can be further improved, and the overall benefits of aquaculture can be enhanced.

### 4.5. Analysis of Differences in Physiological Indexes of Different Stocks of Litopenaeus vannamei

In this study, the physiological indicators of *L. vannamei*, including total antioxidant capacity (T-AOC), superoxide dismutase (SOD) activity, and catalase (CAT) activity, showed no significant differences between the groups. This indicates that the environmental and physiological conditions of the shrimp in each group were largely consistent throughout the experiment, without significant environmental stress or other external disturbances. This observation suggests that the farming conditions provided during the experiment did not induce oxidative stress responses or significant physiological pressure, thus ensuring the consistency of the experimental conditions. Antioxidant enzymes, such as SOD and CAT, are key indicators used to assess the antioxidant capacity of organisms and are typically employed to evaluate the response to oxidative stress [50]. These enzymes help neutralize reactive oxygen species (ROS) to prevent oxidative damage, thereby maintaining the redox balance within cells [51]. T-AOC, as a comprehensive indicator of antioxidant capacity, reflects the overall antioxidant status of the organism [52]. In this study, the stable activity of these antioxidant enzymes between the groups indicates that the shrimp populations did not experience significant oxidative stress throughout the experiment. This result further confirms the controlled and consistent experimental conditions. Since oxidative stress is often associated with environmental stress, stocking density, or pathological conditions, the stability in antioxidant enzyme activity rules out these factors as potential influences on the experimental results. In particular, in aquaculture, changes in antioxidant indicators are often early signs of stress response. The absence of such changes in this study indicates that the experimental conditions were relatively ideal and stable for the shrimp populations.

Moreover, the findings regarding antioxidant enzyme activity provide important insights into explaining the observed differences in fatty acid composition. Although there were differences in fatty acid composition among the groups, these differences cannot be attributed to oxidative stress or physiological pressure. Lipid peroxidation is a common cause of changes in fatty acid composition. However, given the stability of antioxidant enzyme activity during the experiment, it is reasonable to infer that the differences in fatty acid composition were likely caused by other factors, such as feed composition or slight variations in farming conditions, rather than by oxidative stress. In summary, the stability of the antioxidant physiological indicators demonstrates that the shrimp populations maintained good health throughout the experiment, without significant environmental or physiological stress. This result provides a solid foundation for the analysis of differences in fatty acid composition, ruling out oxidative stress as a potential factor influencing changes in fatty acids.

### 4.6. Analysis of Differences in Genetic Diversity of Different Stocks of Litopenaeus vannamei

Genetic diversity is the cornerstone of a species’ ability to adapt to intricate and fluctuating environments, ensuring survival and evolution [53]. The extent of genetic variation significantly impacts a species’ evolutionary capacity and its resilience to environmental shifts. Parameters such as Polymorphic Information Content (PIC) are vital in assessing populations’ genetic diversity [54]. PIC represents the probability that alleles from offspring and their parents originate from the same source. PIC values exceeding 0.5 are considered highly polymorphic, while values between 0.25 and 0.5 are moderately polymorphic, and values below 0.25 are categorized as low polymorphism. In the study of the *L. vannamei* populations, the PIC values for the LvA, LvB, and LvC populations were 0.303 ± 0.125, 0.317 ± 0.124, and 0.300 ± 0.130, respectively, indicating moderate polymorphism levels across the three populations.

The single nucleotide polymorphism (SNP) density and the nucleotide diversity (π) for all three populations were low, suggesting limited genetic diversity. The observed heterozygosity (Ho) was consistent in the LvA and LvB populations (0.048 ± 0.001), but significantly lower in the LvC population (0.033 ± 0.014), indicating a higher degree of inbreeding in LvC. This was further confirmed by the inbreeding coefficient (FIS), which was highest in the LvC population (0.887), underscoring the limited genetic diversity issue. Analysis of the genetic differentiation index revealed a certain degree of genetic differentiation among the populations. The genetic differentiation coefficient (FST = 0.106) and genetic distance (DR = 0.112) between the LvB and LvC populations were the highest, indicating significant genetic differences between these two populations. This is consistent with the results of Ren et al. (FST = 0.09) [55]. Comparatively, the genetic differentiation between LvA and the other two populations was lower but still notable. Gene flow (Nm) data indicated that gene flow was highest between LvA and LvB (4.214), suggesting frequent genetic exchange between these two populations. Conversely, gene flow between LvC and the other two populations was lower, with the lowest gene flow observed between LvC and LvB (2.108), further supporting the conclusion of limited genetic diversity in the LvC population. These findings highlight the need to strengthen genetic monitoring of *L. vannamei* populations to ensure the diversity of genetic resources and the maintenance of population health, especially in the face of dual environmental and economic pressures. Through the effective management and use of these genetic data, we can lay a solid foundation for future breeding activities. Scholars Crawford and Littlepohn [56] emphasize that differentiation time predominantly influences inter-population variations, with genetic distance serving as an objective metric to gauge differentiation time and genetic diversity within populations. In this study, the populations with the most significant genetic distance were LvB and LvC, with a measured value of 0.112. In contrast, the genetic distance between LvA and LvB was the smallest, at 0.057, indicating a close genetic relationship between LvA and LvB. At the same time, LvB and LvC exhibited a distant genetic relationship. This study revealed significant genetic diversity and structure differences among the three *L. vannamei* populations. The LvC population exhibited lower genetic diversity and a higher inbreeding coefficient, indicating genetic uniformity and a potentially higher risk of inbreeding depression. Lower genetic diversity may reduce the population’s ability to adapt to environmental changes, leading to weaker resilience and long-term cultivation performance [57]. To enhance the genetic diversity of these populations, future breeding programs should increase gene flow between populations. Additional genetic resources can increase genetic variation and improve inbreeding conditions, ensuring the health and sustainability of cultured *L. vannamei* populations.

## 5. Conclusions

This study presents a comprehensive comparative analysis of the phenotypic characteristics, nutritional composition, physiological biochemistry, and genetic diversity among three populations of *L. vannamei*. The results revealed no significant differences in muscle crude protein content, fat content, or amino acid content among the three populations, indicating a high degree of consistency and substantial nutritional value. Principal component analysis identified body length, carapace length, and the length of the second antenna as critical distinguishing features among these populations, with a cumulative contribution rate of 70.20%. Genetic diversity analysis showed that the LvC population exhibited a higher inbreeding coefficient and lower observed heterozygosity, highlighting issues of constrained genetic diversity. Furthermore, the genetic differentiation coefficient and genetic distance indicated the most significant genetic differentiation between LvB and LvC, whereas LvA exhibited higher gene flow with the other two populations, suggesting more frequent genetic exchanges. In summary, this study found no significant differences in nutritional composition or morphological traits among the three populations but did identify significant differences in genetic diversity. These findings underscore the importance of enhancing gene flow between populations and optimizing breeding strategies to improve genetic diversity and adaptability. Such measures are crucial for ensuring the sustainable development of *L. vannamei*.

## Figures and Tables

**Table 1:** Information on samples of *Litopenaeus vannamei*.

Survey Point	Sample Name	Quantities	Average Weight (g)	Average Armor Length (mm)	Month	Water Temperature (°C)	Salinity (ppt)	Cultivation Environment
Maoming City	LvA	30	59.28 ± 6.39	143.47 ± 3.12	3 to 4	27.5	30	Natural bottom ponds
Zhanjiang City	LvB	30	60.01 ± 6.63	142.87 ± 2.50	3 to 4	27.4	29	Concrete bottoms
Yangjiang City	LvC	30	60.32 ± 8.94	145.17 ± 4.32	3 to 4	27.4	30	Canvas bottoms

**Table 2:** Results of analysis of variance (ANOVA) for eight quantifiable traits in three groups of *Litopenaeus vannamei*.

Variant	LvA	LvB	LvC
Body length/second step foot length	6.0533 ± 0.3866 ^ab^	6.1767 ± 0.32129 ^a^	5.9167 ± 0.35631 ^b^
Body length/basal distance of fifth footsteps	11.9667 ± 1.0083 ^a^	11.9533 ± 0.6622 ^a^	12.0300 ± 0.84431 ^a^
Body length/cephalothoracic armor length	3.7500 ± 0.2418 ^ab^	3.8867 ± 0.1717 ^a^	3.6000 ± 0.2084 ^b^
Body length/frontal horn length	8.5767 ± 0.76954 ^ab^	8.9167 ± 0.6097 ^a^	8.5033 ± 0.7573 ^b^
Frontal horn length/cephalothoracic armor length	0.4367 ± 0.4901 ^a^	0.4367 ± 0.4901 ^a^	0.4267 ± 0.4498 ^a^
First whip length/cephalothoracic armor length	1.5733 ± 0.2586 ^a^	1.6467 ± 0.2713 ^a^	1.5000 ± 0.31948 ^a^
Second whip length/cephalothoracic armor length	0.2900 ± 0.10939 ^a^	0.4267 ± 0.06400 ^b^	0.3967 ± 0.0556 ^b^
First whip length/body length	0.4167 ± 0.6989 ^a^	0.4200 ± 0.0610 ^a^	0.4067 ± 0.0785 ^a^
Second whip length/body length	0.0800 ± 0.4068 ^a^	0.100 ± 0.0000 ^b^	0.1000 ± 0.0000 ^b^

Note: Superscript letters (a, b, ab) within the same row denote statistically significant differences between groups. Values sharing the same letter are not significantly different (*p* > 0.05).

**Table 3:** Characteristic vectors of eight traits of three populations of *Litopenaeus vannamei* on three principal components and the contribution of the principal components.

Morphological Proportionality Traits	Principal Component		
	1	2	3
Body length/second step foot length	0.339	0.012	−0.118
Body length/basal distance of fifth footsteps	0.214	−0.049	−0.060
Body length/cephalothoracic armor length	0.286	0.088	−0.053
Body length/frontal horn length	0.301	−0.057	0.118
Frontal horn length/cephalothoracic armor length	−0.113	0.176	−0.248
First whip length/cephalothoracic armor length	0.035	0.485	0.009
Second whip length/cephalothoracic armor length	0.010	0.047	0.463
First whip length/body length	−0.048	0.482	0.003
Second whip length/body length	−0.163	0.010	0.555
Contribution of each principal component (%)	31.756	21.383	17.065
Cumulative contribution (%)	31.756	53.139	70.204

**Table 4:** Amino acid composition and content (g/100 g, wet weight) of muscle from three *Litopenaeus vannamei* groups.

Amino Acids	LvA	LvB	LvC
Aspartic acid Asp ^@^	1.54 ± 0.09 ^a^	1.53 ± 0.04 ^a^	1.50 ± 0.05 ^a^
Threonine Thr *	0.60 ± 0.03 ^a^	0.59 ± 0.01 ^a^	0.57 ± 0.02 ^a^
Serine Ser	0.53 ± 0.03 ^a^	0.53 ± 0.01 ^a^	0.50 ± 0.02 ^a^
Glutamic acid Glu ^@^	2.43 ± 0.15 ^a^	2.45 ± 0.06 ^a^	2.45 ± 0.06 ^a^
Glycine Gly ^@^	1.04 ± 0.06 ^a^	1.08 ± 0.03 ^a^	1.04 ± 0.03 ^a^
Alanine Ala ^@^	0.96 ± 0.04 ^a^	0.97 ± 0.02 ^a^	0.96 ± 0.03 ^a^
Cystine Cys	0.22 ± 0.02 ^a^	0.17 ± 0.02 ^b^	0.16 ± 0.01 ^b^
Val *	0.73 ± 0.04 ^a^	0.73 ± 0.02 ^a^	0.70 ± 0.02 ^a^
Methionine Met *	0.41 ± 0.02 ^a^	0.38 ± 0.01 ^b^	0.35 ± 0.01 ^b^
Isoleucine IIe *	0.66 ± 0.03 ^a^	0.65 ± 0.01 ^a^	0.63 ± 0.02 ^a^
Leucine Leu *	1.25 ± 0.07 ^a^	1.22 ± 0.03 ^a^	1.18 ± 0.04 ^a^
Tyr tyrosine	0.48 ± 0.02 ^a^	0.45 ± 0.01 ^a^	0.41 ± 0.01 ^b^
Phenylalanine Phe *	0.67 ± 0.03 ^a^	0.60 ± 0.01 ^b^	0.61 ± 0.02 ^b^
Lys *	1.21 ± 0.06 ^a^	1.20 ± 0.03 ^a^	1.20 ± 0.03 ^a^
Histidine His ^&^	0.33 ± 0.02 ^a^	0.32 ± 0.01 ^a^	0.31 ± 0.01 ^a^
Arginine Arg ^&^	0.95 ± 0.05 ^a^	0.95 ± 0.03 ^a^	0.91 ± 0.03 ^b^
Proline Pro	1.27 ± 0.08 ^a^	1.30 ± 0.06 ^a^	1.25 ± 0.06 ^a^
EAA	5.86 ± 0.31 ^a^	5.70 ± 0.13 ^b^	5.55 ± 0.16 ^c^
SEAA	1.28 ± 0.07 ^a^	1.27 ± 0.04 ^a^	1.22 ± 0.03 ^a^
NEAA	8.13 ± 0.47 ^a^	8.17 ± 0.21 ^a^	7.97 ± 0.28 ^b^
DAA	5.97 ± 0.34 ^a^	6.04 ± 0.15 ^a^	5.95 ± 0.17 ^a^
TAA	15.27 ± 0.84 ^a^	15.13 ± 0.38 ^a^	14.73 ± 0.47 ^a^
EAA/TAA	0.38 ± 0.00 ^a^	0.37 ± 0.00 ^a^	0.37 ± 0.00 ^a^
EAA/NEAA	0.72 ± 0.00 ^a^	0.70 ± 0.00 ^a^	0.70 ± 0.00 ^a^

Note: * indicates essential amino acids; ^@^ indicates flavoring amino acids; ^&^ indicates semi-essential amino acids; EAA: total essential amino acids; SEAA: total semi-essential amino acids; NEAA: total non-essential amino acids; DAA: total flavoring amino acids. Superscript letters (a, b, c) within the same row indicate statistically significant differences between groups (*p* < 0.05). Values sharing the same letter are not significantly different.

**Table 5:** Fatty acid composition and content of muscle fatty acids (mg/100 g, wet weight) of three groups of *Litopenaeus vannamei*.

Fatty Acids	LvA	LvB	LvC
Palmitic acid C16:0	168.30 ± 2.69 ^a^	138.53 ± 15.23 ^b^	148.87 ± 9.56 ^ab^
Palmitoleic acid C16:1	5.77 ± 0.12 ^a^	4.10 ± 0.60 ^b^	5.13 ± 0.42 ^c^
C17:0 heptadecanoic acid	10.67 ± 0.12 ^a^	9.03 ± 1.03 ^b^	9.90 ± 0.56 ^ab^
Stearic acid C18:0	126.43 ± 2.27 ^a^	109.67 ± 13.27 ^b^	114.70 ± 6.42 ^b^
Oleic acid C18:1 n9c	123.07 ± 2.04 ^a^	106.27 ± 13.55 ^b^	104.53 ± 3.40 ^b^
Linoleic acid C18:2n6c	160.57 ± 2.57 ^a^	138.77 ± 17.04 ^b^	140.23 ± 8.30 ^b^
Arachidic acid C20:0	7.80 ± 0.10 ^a^	5.13 ± 4.46 ^b^	7.40 ± 0.40 ^a^
Linolenic acid C18:3n3	9.33 ± 0.15 ^a^	7.57 ± 0.97 ^b^	7.70 ± 0.60 ^b^
Eicosatetraenoic acid C20:1	6.60 ± 0.10 ^a^	6.07 ± 0.87 ^a^	6.07 ± 0.55 ^a^
Eicosadienoic acid C20:2	15.33 ± 0.40 ^a^	14.03 ± 1.68 ^a^	14.40 ± 0.87 ^a^
Docosanoic acid C22:0	9.53 ± 0.15 ^a^	8.60 ± 1.61 ^a^	8.73 ± 0.49 ^a^
Arachidonic acid ARAC20:4n6	31.57 ± 0.45 ^a^	27.17 ± 3.63 ^b^	28.97 ± 1.46 ^b^
Erucic acid C22:1n9	3.90 ± 0.00 ^a^	4.37 ± 0.42 ^b^	6.47 ± 0.45 ^b^
XXIII carbonic acid C23:0	1.12 ± 1.92 ^a^	1.13 ± 1.96 ^a^	1.13 ± 1.96 ^a^
Eicosapentaenoic acid C20:5n3(EPA)	54.80 ± 0.92 ^a^	47.37 ± 4.97 ^b^	51.10 ± 2.97 ^ab^
C22:6n3(DHA)	60.30 ± 0.95 ^a^	54.93 ± 5.51 ^b^	60.30 ± 3.06 ^a^
Total fatty acid content TOTAL	793.97 ± 12.94 ^a^	682.73 ± 85.68 ^b^	715.63 ± 42.04 ^c^
Total saturated fatty acids ΣSFA	317.83 ± 5.32 ^a^	267.17 ± 36.44 ^b^	285.97 ± 19.03 ^b^
Monounsaturated fatty acids ΣMUFA	10.50 + 0.10 ^b^	10.43 + 1.25 ^b^	12.53 + 0.76 ^a^
Polyunsaturated fatty acids ΣPUFA	277.10 ± 4.49 ^a^	242.42 ± 28.63 ^b^	251.60 ± 13.75 ^b^
DHA ± EPA	115.10 ± 1.87 ^a^	103.30 ± 10.47 ^b^	111.40 ± 5.86 ^a^
n-3 series polyunsaturated fatty acidsn-3ΣPUFA	124.43 ± 2.02 ^a^	109.87 ± 11.43 ^b^	119.10 ± 6.46 ^b^
n-6 series polyunsaturated fatty acidsn-6ΣPUFA	192.13 ± 3.01 ^a^	165.93 ± 20.67 ^b^	169.20 ± 9.42 ^b^

Note: Superscript letters (a, b, ab, c) within the same row indicate statistically significant differences between groups (*p* < 0.05). Values sharing the same letter are not significantly different.

**Table 6:** Statistics on population genetic diversity parameters of three populations of *Litopenaeus vannamei*.

Population	SNP Number	SNP Density (SNP/Kb)	Nucleotide Diversity (π)	Polymorphism Information Content (PIC)	Observed Heterozygosity (Ho)	Inbreeding Coefficient (FIS)
LvA	29047925	17.461	3.11 × 10^−5^	0.303 ± 0.125	0.048 ± 0.001	0.835
LvB	25491738	15.323	2.98 × 10^−5^	0.317 ± 0.124	0.048 ± 0.001	0.834
LvC	26677995	16.036	15.84 × 10^−5^	0.300 ± 0.130	0.033 ± 0.014	0.887

**Table 7:** Genetic differentiation index (FST), inter-population genetic distance (DR), and gene flow (Nm) statistics of *Litopenaeus vannamei*.

Population 1	Population 2	Population Differentiation Coefficient (FST)	Genetic Distance (DR)	Gene Flow (Nm)
LvA	LvB	0.056	0.057	4.214
LvA	LvC	0.084	0.088	2.726
LvB	LvC	0.106	0.112	2.108

## Data Availability

No new data were created or analyzed in this study.
